# Supplementary material for: Psychophysiological Effects of Lactobacillus plantarum PS128 in Patients with Major Depressive Disorder: A Preliminary 8-Week Open Trial
Source: Nutrients. 2021 Oct 22;13(11):3731. doi: 10.3390/nu13113731 (PMC8618644; doi:10.3390/nu13113731)
Supplement: Supplementary file 1 [file nutrients-13-03731-s001.zip › Supplementary materials/nutrients-1416656_Supplemental_Figures.pdf]

Supplemental Information

Psychophysiological Effects of *Lactobacillus Plantarum* PS128 in Patients with Major Depressive Disorder: A Preliminary 8-Week Open Trial

Hui-Mei Chen[1], Po-Hsiu Kuo[1,2,*], Chia-Yueh Hsu[3,4], Yi-Hung Chiu[3], Yen-Wenn Liu[5], Mong-Liang Lu[3,4] , Chun-Hsin Chen[3,4,*]

[1]Institute of Epidemiology and Preventive Medicine, National Taiwan University, Taipei100, Taiwan
[2]Department of Psychiatry, National Taiwan University Hospital, Taipei 100, Taiwan
[3]Department of Psychiatry, Taipei Municipal Wan-Fang Hospital, Taipei Medical University, Taipei 116, Taiwan
[4]Department of Psychiatry, School of Medicine, College of Medicine, Taipei Medical University, Taipei 110, Taiwan
[5]Institute of Biochemistry of Molecular Biology, National Yang-Ming University, Taipei 112, Taiwan

[*]Correspondence: phkuo@ntu.edu.tw (P.-H.K.); chunhsin57@gmail.com (C.-H.C.); Tel.: +886-2-3366-8015 (P.-H.K.); +886-2-2930-7930 (ext. 53961) (C.-H.C.); Fax: +886-2-2351-1955 (P.-H.K.); +886-2-29335221 (C.-H.C.)

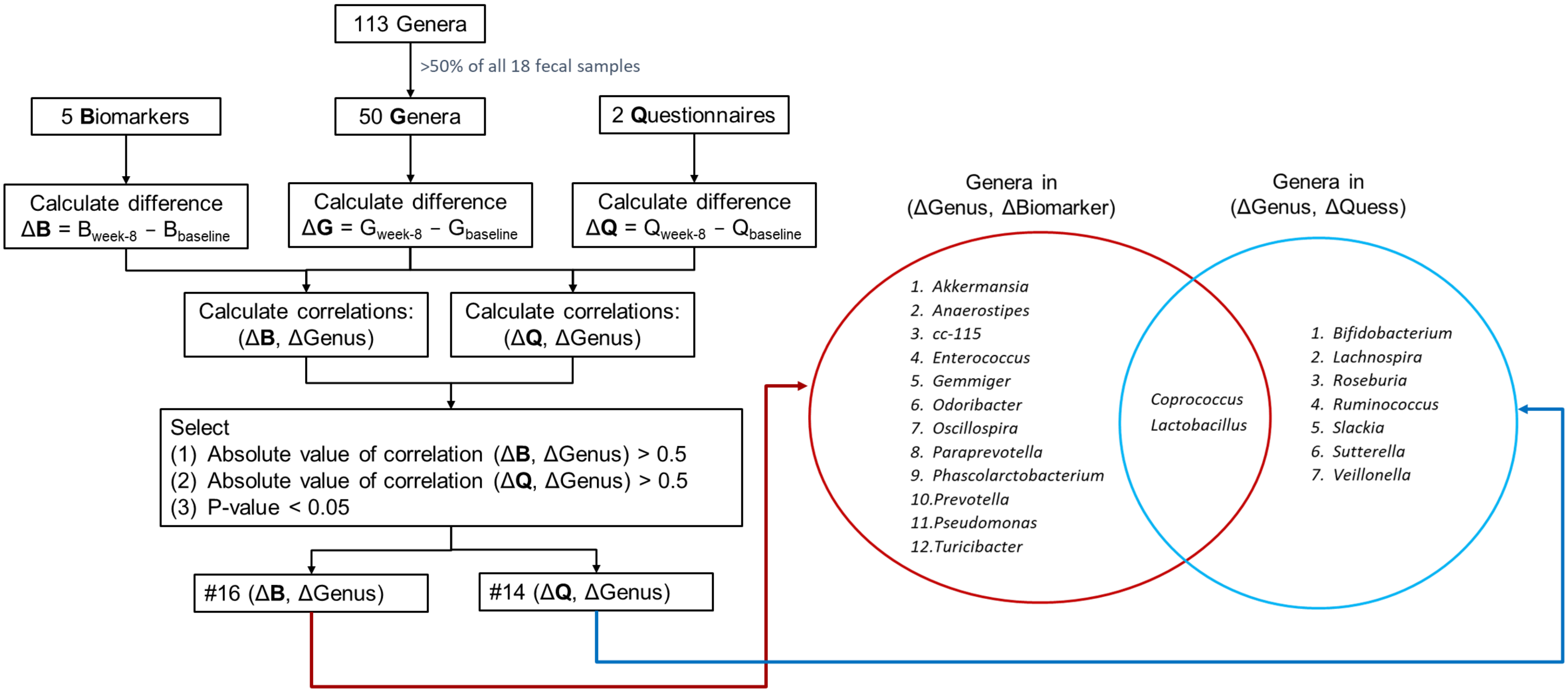

Figure S1. Flow chart of selection process. In total of 113 general were detected within fecal samples. First, the genera which were detected in at least 50% of all fecal samples were selected. Second, the differences between baseline and week 8 were calculated in three separate datasets: the relative abundance of genera, the biomarker levels, and the questionnaire scores. The difference datasets were denoted by ΔB for biomarkers, ΔG for genera, and ΔQ for questionnaires. Third, correlations of difference values (Δ) between the three difference datasets were evaluated using the Spearman correlation coefficient with the Wilcoxon rank sum test. Fourth, correlated pairs with an absolute value of correlation coefficient higher than 0.5 with p-values less than 0.005 were selected. Finally, genera which appeared in more than one significant pair were only included once within the final Venn diagram

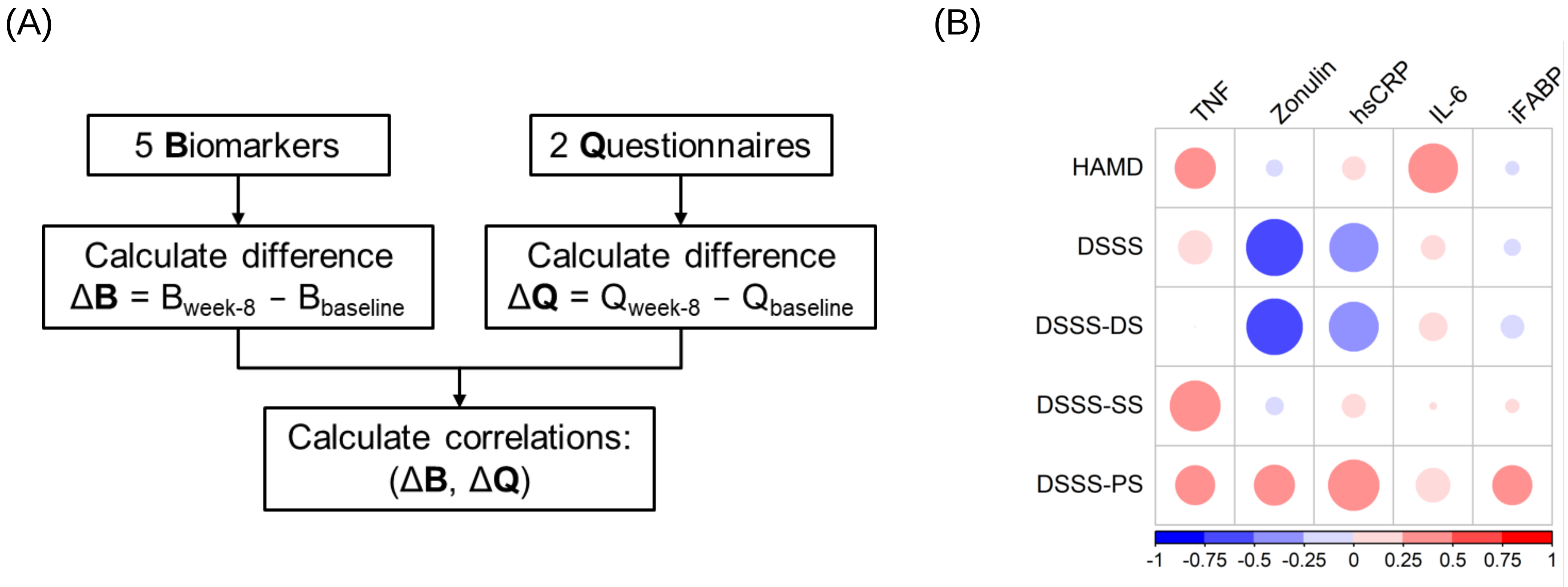

Figure S2. (A) Flow chart of correlation analysis between biomarkers and questionnaire scores. The differences between baseline and week 8 were calculated in two datasets: the biomarker levels and the questionnaire scores. The difference datasets were denoted by ΔB for biomarkers and ΔQ for questionnaires. Subsequently, correlations of difference values (Δ) between the two difference datasets was evaluated using the Spearman correlation coefficient with the Wilcoxon rank sum test. **(B) The correlations between inflammatory biomarkers and depressive assessments.** Red denotes positive correlation; blue denotes negative correlation. The column names are five inflammatory biomarkers: tumor necrosis factor alpha (TNF), Zonulin, high-sensitive C-Reactive Protein (hsCRP), interleukin6 (IL-6), and intestinal fatty acid binding protein (I-FABP). The row names are severity symptom measurements: HAMD-17 and Depression and Somatic Symptoms Scale (DSSS). DS means the depression subscale; SS means the somatic subscale; PS means pain subscale. They are all subscales of DSSS.

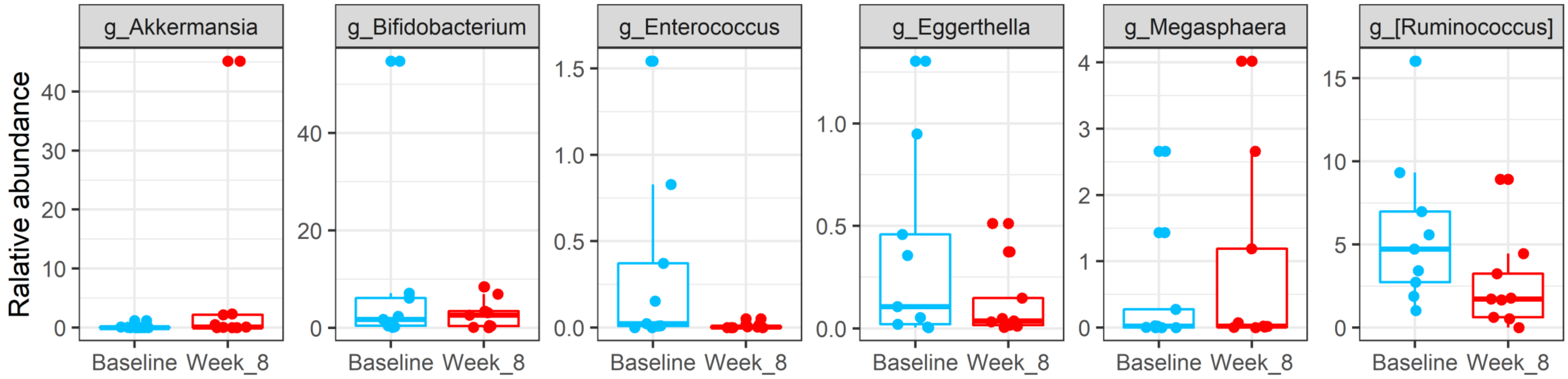

Figure S3. Box plots of the relative abundance of six genera. The six top genera were selected according to the results of the rank of the change of relative abundances or that of statistical testing p-values between the two time points.
